# Volume electron microscopy reveals age-related circuit remodeling in the auditory brainstem

**DOI:** 10.3389/fncel.2022.1070438

**Published:** 2022-12-15

**Authors:** Daniela Chequer Charan, Yunfeng Hua, Haoyu Wang, Wenqing Huang, Fangfang Wang, Ana Belén Elgoyhen, Kevin M. Boergens, Mariano N. Di Guilmi

**Affiliations:** ^1^Instituto de Investigaciones en Ingeniería Genética y Biología Molecular, Dr. Héctor N. Torres, INGEBI-CONICET, Buenos Aires, Argentina; ^2^Shanghai Institute of Precision Medicine, Ninth People’s Hospital, Shanghai Jiao Tong University School of Medicine, Shanghai, China; ^3^Department of Physics, The University of Illinois at Chicago, Chicago, IL, United States

**Keywords:** calyx of Held, aging, large-scale 3D electron microscopic, MNTB, brainstem

## Abstract

The medial nucleus of the trapezoid body (MNTB) is an integral component of the auditory brainstem circuitry involved in sound localization. The giant presynaptic nerve terminal with multiple active zones, the calyx of Held (CH), is a hallmark of this nucleus, which mediates fast and synchronized glutamatergic synaptic transmission. To delineate how these synaptic structures adapt to reduced auditory afferents due to aging, we acquired and reconstructed circuitry-level volumes of mouse MNTB at different ages (3 weeks, 6, 18, and 24 months) using serial block-face electron microscopy. We used C57BL/6J, the most widely inbred mouse strain used for transgenic lines, which displays a type of age-related hearing loss. We found that MNTB neurons reduce in density with age. Surprisingly we observed an average of approximately 10% of poly-innervated MNTB neurons along the mouse lifespan, with prevalence in the low frequency region. Moreover, a tonotopy-dependent heterogeneity in CH morphology was observed in young but not in older mice. In conclusion, our data support the notion that age-related hearing impairments can be in part a direct consequence of several structural alterations and circuit remodeling in the brainstem.

## Introduction

Hearing loss (HL) is a multifactorial progressive symptom with a high prevalence in society and with a projected increased impact for the next decades^1^ (Elgoyhen, 2022). Different environmental insults such as continuous exposure to high levels of noise, pharmacological treatments with ototoxic compounds, as well as some dietary components, are just a few of the environmental factors that lead to HL, in addition to genetic causes (Haile et al., 2021). Furthermore, aging, with its slowed or limited recovery from tissue damage, contributes to the high occurrence of auditory impairments in older people (Collins, 1997). In this sense, age-related hearing loss (ARHL, also known as presbycusis) is a disability with high prevalence (Gates and Mills, 2005; Fu et al., 2010; Lauer et al., 2019) and one of the most important factors leading to cognitive decline and dementia (Deal et al., 2018; Loughrey et al., 2018; Livingston et al., 2020). It is widely accepted that pathological alterations in the peripheral auditory organ and the consequent changes in central plasticity are the mechanisms underlying the ARHL (Frisina and Walton, 2006; Caspary et al., 2008; Hughes et al., 2010; Parthasarathy and Kujawa, 2018; Wang M. et al., 2021). Although cochlear deafferentation (synaptopathy) is an early contributor to age-related auditory decline (Sergeyenko et al., 2013; Parthasarathy and Kujawa, 2018; Boero et al., 2020), the central alterations in multiple brainstem nuclei (Casey and Feldman, 1982; Singh et al., 2018; Vicencio-Jimenez et al., 2021), as well as in the auditory cortex (Hughes et al., 2010), are less studied. Moreover, ARHL is frequently accompanied by a deterioration in sound localization (Burke et al., 2022; Xiong et al., 2022), an important auditory function of the brainstem (Masterton et al., 1967).

The medial nuclei of the trapezoid body (MNTB), located in the superior olivary complex, is one of the first auditory relays involved in sound localization (Borst and Soria van Hoeve, 2012). As other auditory structures, the MNTB is topographically organized along a mediolateral axis, where high frequencies (HF) are represented medially and low frequencies (LF) laterally (Sommer et al., 1993). The synapse formed by the principal neurons (PNs) of the MNTB and its presynaptic terminal, the so-called calyx of Held (CH), is one of the most studied synapse models (Baydyuk et al., 2016). The large size of the CH enables paired patch-clamp recording on both the nerve terminal and its postsynaptic target (Schneggenburger and Forsythe, 2006), allowing the study of synaptic transmission. In addition to the large excitatory (glutamatergic) calyceal input, MNTB neurons also receive non-calyceal excitatory inputs (Hamann et al., 2003) and somatic inhibitory inputs from different origins (Awatramani et al., 2004), making it an ideal model system to study neural circuit development (Holcomb et al., 2013), as well as neural code integration. Electrophysiological studies of the MNTB are oftentimes complemented by gross morphological analysis using optical approaches [e.g., intracellular calcium sensitive dyes (Helmchen et al., 1997), genetically encoded fluorophores (Kronander et al., 2018), and dextran dyes conjugated with fluorophores (Grande and Wang, 2011)]. Since its first description by Held (1893), a number of studies, primarily using electron microscopy (EM), have been carried out in the MNTB (Lenn and Reese, 1966; Nakajima, 1971; Jean-Baptiste and Morest, 1975; Rowland et al., 2000; Sätzler et al., 2002; Taschenberger et al., 2002). These studies aimed at quantitatively studying the cellular architecture of the CH, including individual active zones (Rowland et al., 2000), synaptic vesicles (Taschenberger et al., 2002), mitochondria (Thomas et al., 2019), and myelin sheath (Sinclair et al., 2017). As previous work mainly focused on rodents at prehearing (P0–P9; Hoffpauir et al., 2006; Holcomb et al., 2013) or juvenile stages (P21–P35, Sinclair et al., 2017; Thomas et al., 2019), knowledge concerning the age-related ultrastructure changes in the MNTB has not been updated since the 80s. These were only performed in random thin sections and small blocks of rat brainstems (Casey and Feldman, 1985).

Making use of the recent advances in automation of serial section EM techniques and artificial intelligence-assisted image segmentation (Bock et al., 2011; Briggman et al., 2011; Kasthuri et al., 2015; Motta et al., 2019), in the present work we acquired large EM volumes of the MNTB covering the whole tonotopic range in a mouse line which displays accelerated hearing loss (C57BL/6J; Mikaelian et al., 1974; Henry and Lepkowski, 1978; Mikaelian, 1979). Structural quantifications of the CH in young (3 weeks), adult (6 months), as well as aging (18 and 24 months) mice revealed age-related changes including a decrease in neuron density, a reduction in CH morphological heterogeneity, and a degradation of the tonotopy-dependent innervation pattern. Both degenerated CHs and MNTB neurons were prevalent in aging animals, with CH disintegration prior to PN degeneration. This observation suggests that the degeneration of the post-synaptic MNTB PN cells occurs as a consequence of the expected deafferentation due to the lack of auditory input.

## Materials and Methods

### Animals

Male C57BL/6J mice of 3 weeks (3 W), 6, 18, and 24 months (6 M, 18 M, and 24 M, respectively) were used in this study. All mice used in this study were the C57BL/6J line originally from Jackson Labs (Bar Harbor, ME). Animals (18 M, 24 M) were supplied by Prof. A Qin (Shanghai Ninth People’s Hospital) and young animals (3 W and 6 M) were purchased from the Shanghai Jihui Laboratory Animal Care Co., Ltd (Shanghai, China). All experimental procedures were approved by the ethical committee of Shanghai Ninth People’s Hospital (No. SH9H-2020-A420-1) and conducted at the Shanghai Institute of Precision Medicine and Ear Institute of Shanghai Jiao Tong University School of Medicine.

### Auditory brainstem response (ABR)

Auditory Brainstem Response (ABR) measurements were conducted as previously described (Wang H. et al., 2021; Zhang et al., 2022). Briefly, animals were anesthetized with isoflurane (2%) inhalation and the body temperature was maintained near 37°C using a regulated heating pad with a thermal probe placed under the abdomen (Homeothermic Monitoring System, Harvard Apparatus, USA). Sound stimuli, which were generated by a SigGen RP unit (Tucker-Davis Tech. Inc., USA), were delivered *via* a speaker (MF1, Tucker-Davis Tech. Inc., USA) placed 10 cm away in front of the animal’s vertex. ABRs were evoked by a ramped cosine-type waveform with 3 ms tone pips (1 ms rise-fall) and were recorded *via* three subdermal needle electrodes placed at the animal’s vertex (active electrode), left infra-auricular mastoid (reference electrode) and right shoulder region (ground electrode). Signal digitalization, acquisition, and routine speaker calibration were done with the BioSigRZ software (Tucker-Davis Tech. Inc., USA). The raw signals were amplified (5,000×) and bandpass filtered (0.03–5 kHz). The sound level of stimulus started from 90 dB SPL and was reduced in 5 dB steps to about 10 dB below threshold (the threshold being defined as the lowest stimulus needed for evoking visible responses).

### Tissue preparation and staining

Animals were anesthetized using isoflurane and perfused transcardially with 0.15 M sodium cacodylate buffer (pH 7.4), followed by a fixation mixture containing 2% paraformaldehyde and 2.5% glutaraldehyde (buffered with 0.08 M sodium cacodylate solution, pH 7.4). An opening was created in the skull by a posterior incision and the brains were kept in the open skull in the fixative solution at 4°C for 48 h. Then the brainstems were dissected carefully and washed in ice-cold 0.15 M cacodylate buffer (pH 7.4). While submerged in the same buffer, the tissue was cut coronally at a thickness of 400 μm using a vibratome (VT 1200S, Leica, Germany). Slices containing the MNTB were anatomically identified under a dissecting microscope and collected after careful removal of the surrounding nerve tissues using a razor blade.

Staining of the MNTB samples was performed as described by Hua et al. (2015) and Gour et al. (2021), with minor modifications. In brief, the samples were washed twice in 0.15 M cacodylate solution (pH 7.4) for 30 min each and sequentially immersed in 2% OsO_4_ (Ted Pella, CA, USA), 2.5% potassium ferrocyanide (Sigma Aldrich, MO, USA), and 2% OsO_4_ at room temperature (RT) for 1.5, 1.5, and 1.0 h, respectively. All solutions were buffered with 0.15 M cacodylate solution (pH 7.4). After being washed in cacodylate (0.15 M, pH 7.4) and nanopore-filtered water for 30 min each, the samples were incubated in filtered 1% thiocarbonhydrazide (Sigma Aldrich, MO, USA) for 1 h at RT, unbuffered OsO_4_ aqueous solution (2%) for 1.5 h, 1% uranyl acetate (Sigma Aldrich, MO, USA) at 50°C for 2 h, and lead aspartate solution (0.03 M, pH 5.0 adjusted by KOH, EMS, USA) at 50°C for 2 h, with intermediate wash steps in nanopore-filtered water (twice, 30 min each). Next, the stained tissues were dehydrated through a graded ethanol series (50%, 75%, 90%, 100%, 30 min each at 4°C), transferred from there into pure acetone (three times, 45 min each at RT) and infiltrated with a 1:1 mixture of acetone and Spurr’s resin (4.1 g ERL 4221, 0.95 g DER 736, 5.9 g NSA and 1% DMAE; Sigma Aldrich, MO, USA) overnight on a rotator at RT. Then the samples were embedded in pure resin for 8 h and cured in a prewarmed oven (70°C) for 72 h. The orientation of the MNTB was confirmed based on the morphological landmark delimited by the middle line (medial region) and the VIII cranial nerve (lateral region).

### Electron microscopy

The resin-embedded samples were mounted on an aluminum metal rivet (Gatan, United Kingdom; Trim2, Leica, Germany) and trimmed down (Leica Trim2) to a block size of about 1.2 mm × 0.8 mm × 0.4 mm (XYZ) with the MNTB at the center. The block faces were created by smoothing, using an ultramicrotome (UC7, Leica, Germany) equipped with a diamond knife (DIATOME Ltd, Nidau, Switzerland) and then coated with a thin layer of carbon using a sputter-coater (Ted Pella, CA, USA, 208C) for quick 2D low-resolution (pixel size: 0.59 μm × 0.59 μm) EM scans (Gemini300, Carl Zeiss, Germany). The orientation and dimension of the MNTB were determined in each sample based on its characteristic soma distribution of large principal neurons. For serial block-face EM imaging, the sample blocks were further trimmed to a smaller block-face (less than 800 μm × 500 μm) and coated with a thin layer of gold (EM ACE600, Leica, Germany). Image acquisition of the MNTB was carried out using a commercial SBEM system (Gemini300, Carl Zeiss, Germany; 3View2XP, Gatan, United Kingdom) in 1-by-4 montage mode with each tile consisting of 7,000 × 10,000 pixels. Imaging parameters were as follows: incident beam energy 2 keV; 15 nm pixel size; pixel dwell time 1.5 μs. Serial sectioning was done at a thickness of 50 nm. The in-plane stitching was done in FIJI (plug-in; Preibisch et al., 2009) and consecutive slices were then aligned using a self-written MATLAB script based on cross-correlation maximum according to the dorso-vental (X), medio-lateral (Y), and rostro-caudal (Z) axis. This resulted in four large EM volumes of (XYZ) 122.5 μm × 541.8 μm × 92.0 μm (3 W), 136.1 μm × 560.8 μm × 77.2 μm (6 M), 125.6 μm × 548.9 μm × 76.0 μm (18 M) and 152.9 μm × 583.1 μm × 92.4 μm (24 M).

### Automated image segmentation

Automated image segmentation was performed with Voxelytics (scalable minds GmbH, Potsdam, Germany; developed in collaboration with Max-Planck-Institute for Brain Research, Dept. of Connectomics). Briefly, a U-Net [modified from (1706.00120 Superhuman Accuracy on the SNEMI3D Connectomics Challenge)^2^] was used to predict voxel-wise affinities from which, subsequently, an initial over-segmentation was generated through a seeded watershed transform. The over-segmentation was then processed using a median-affinity-based hierarchical agglomeration to produce neuron segmentation (adapted from Funke et al., 2019). Note that this automatic detection algorithm worked well for 3 W, 6 M, and 18 M but not for 24 M, due to the large number of degenerated structures present at this age (see Figure 6). For this reason, we decided to consider only the first three ages for quantification and left the 24 M database just to illustrate the cell density drop, the degree of degeneration and the poly-innervation.

**Figure 1:**
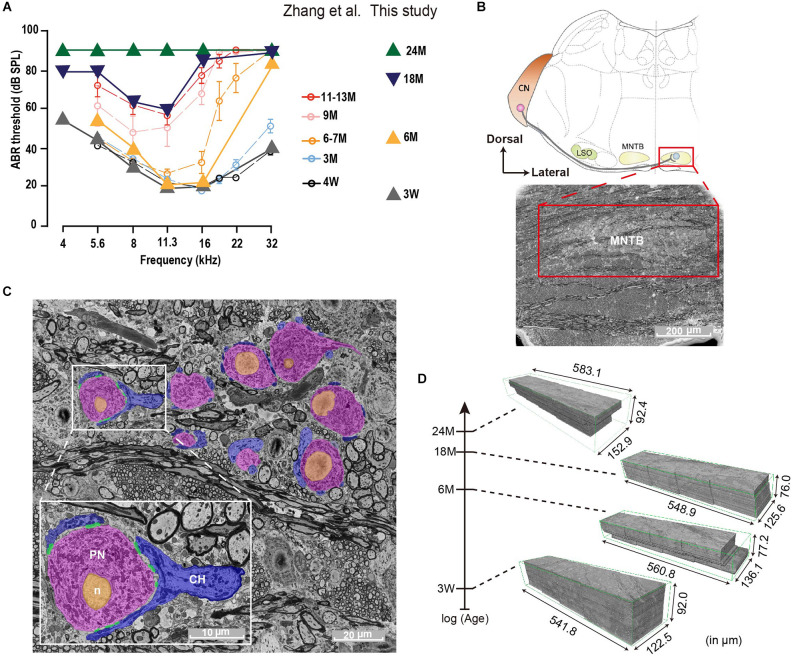
Auditory Brainstem Response (ABR) and SBEM imaging of mouse MNTBs at different ages. **(A)** Average ABR thresholds of C57BL/6J mice at different ages. Plots adapted from Zhang et al. (2022); (open circles) and animals used in this study (triangles) exhibit comparable hearing function at each corresponding age group. Error bars indicate mean ± SEM. **(B)** Top: schematic illustration of the mammalian auditory brainstem. The medial nuclei of the trapezoid body (MNTB) neuron (light blue) receives a monosynaptic projection from a globular bushy cell (red) in the contralateral cochlear nucleus (CN; LSO: lateral superior olive). Bottom: scanning electron micrograph of a brainstem coronally sliced and stained with heavy metals. The MNTB (red box) appears less dark due to densely packed neural cell bodies. Scale bar 200 μm. **(C)** High-resolution EM image of MNTB principal neurons (PN, pink), which are wrapped by calyx nerve endings of (CH, blue). Scale bar 20 μm. Inset: magnification of a representative image showing one calyx terminal forming multiple active zones (AZs; green) onto the cell body of a MNTB neuron. Orange indicates the nucleus (n). Scale bar 10 μm. **(D)** Four SBEM volumes of similar dimensions were acquired from brainstem coronal slices of 3-week, 6-month, 18-month, and 24-month animals, whose ABR thresholds are shown in **(A)**.

**Figure 2:**
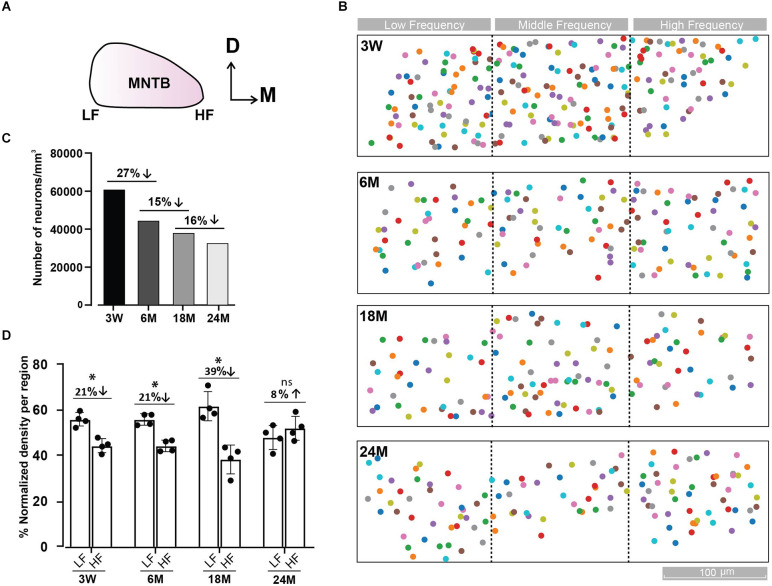
Tonotopic distribution of MNTB cells at different ages. **(A)** Representative scheme of the MNTB showing the dorso (D)—ventral and medio (M) —lateral axes. The high frequency (HF) region is located in the medial and the low frequency (LF) in the lateral side of the MNTB. **(B)** Distribution of MNTB neurons along the tonotopic axis at different ages. Color boxes represent MNTB cells. Dashed black lines delimit the three MNTB regions (lateral, media, and medial). **(C)** Density of MNTB neurons over the whole dataset for each age expressed as the total number of neurons/mm^3^. **(D)** Percentage of neuronal density by region of MNTB cells normalized to the total density of the LF and HF regions. A cube of (XYZ): 70 μm × 70 μm × 70 μm; 0.000343 mm^3^, was randomly placed four times in each region and the number of cells was averaged. The maximal cell density drop between the lateral and medial region occurs at 18 M. Error bars indicate mean ± SEM. *p < 0.05. Up and Down arrows indicate a decrease or increase of the right versus left condition.

**Figure 3:**
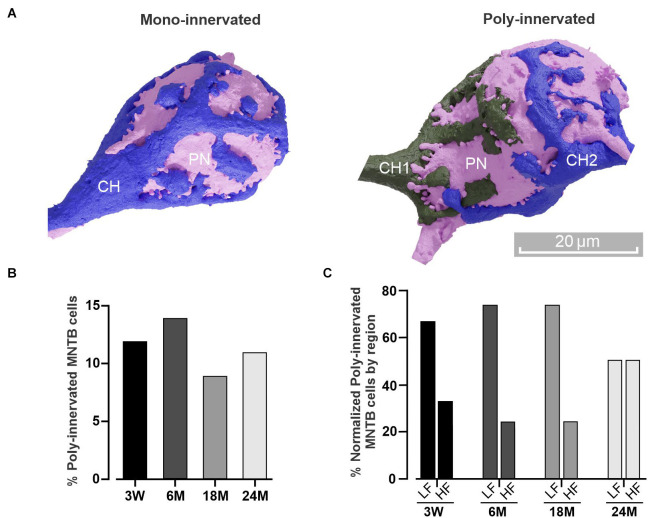
Poly-innervation of MNTB cells is present beyond the critical period. **(A)** Three-dimensional reconstruction of a principal neuron (PN, pink) and its presynaptic (calyx of Held, CH) terminal in the case of a mono-innervated (blue, left) or poly-innervated (blue and gray, right) MNTB cell. Scale bar: 20 μm. **(B)** Percentage of poly-innervated cells at different ages. Note that the value (about 10%) is conserved throughout the analyzed lifespan. **(C)** Percentage of poly-innervated cells at different ages, comparing the HF and LF regions. Note that the LF displays higher poly-innervated cells than the HF.

**Figure 4:**
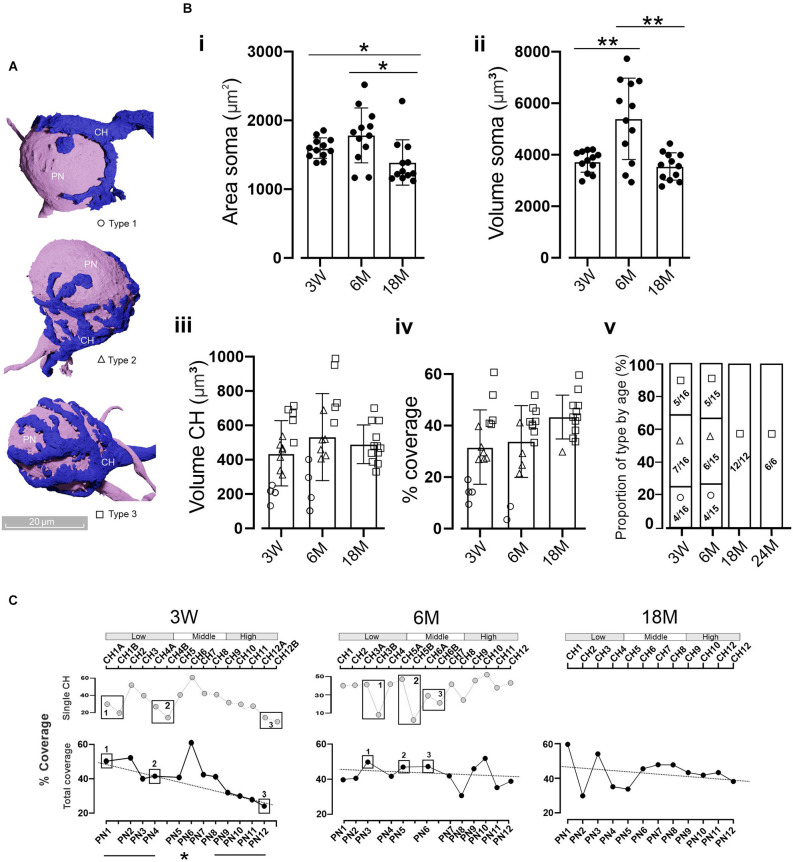
Coverage area of MNTB cells by the CH. **(A)** Three-dimensional reconstructions of MNTB cells showing the heterogeneity in the gross morphology of mature calyces of Held. Calyces with simple (Type 1, circles), medium (Type 2, triangles), or complex structure (Type 3, boxes) were defined according to their cover area (see text). **(B)** Quantification of the PN **(i)** area and volume **(ii)**. Asterisks denote statistically significant differences. Area: 3 M vs. 18 M (*n* = 12; Wilcoxon, *p* = 0.03) and 6 M vs. 18 M (*n* = 12; Wilcoxon, *p* = 0.03). Volume: 3 W vs. 6 M (*n* = 12; Wilcoxon, *p* = 0.012) and 6 M vs. 18 M (*n* = 12, Wilcoxon, *p* = 0.006). Quantification of the CH volume **(iii)** and the PN coverage area **(iv)** displayed no significant differences between ages (*n* = 12, Kruskal-Wallis, *p* = 0.92 and *n* = 12, Kruskal-Wallis, *p* = 0.12, respectively). Note that complex calyces (Type 3) displayed larger volumes and coverage areas than simple ones (Type 1). The proportion of calyx types by age **(v)** displayed a clear presence of complex structures in older animals (chi-square, *p* = 0.0016). Error bars indicate mean ± SEM. **(C)** Percentage of the coverage area along the tonotopic axes considering each individual CH contacting a PN (top panel). In the case of poly-innervated cells (boxes), the coverage area was summed up reaching the total covered area per each PN (bottom panel). Note that, for example, at 3 W PN1 is poly-innervated by CH1A and CH1B, whereas PN2 is mono-innervated by CH2. Despite the presence of poly-innervated MNTB cells at 18 M (Figure 3B), all randomly selected cells displayed mono-innervation. At 3 W there was a linear correlation between the covered area and the tonotopic axes (*R*^2^ = 0.93) with higher values at the LF region (45.4 ± 2.9%) and smaller in the HF (28.1 ± 1.7%; *n* = 4, Wilcoxon, *p* = 0.02). *p < 0.05; **p < 0.005.

**Figure 5:**
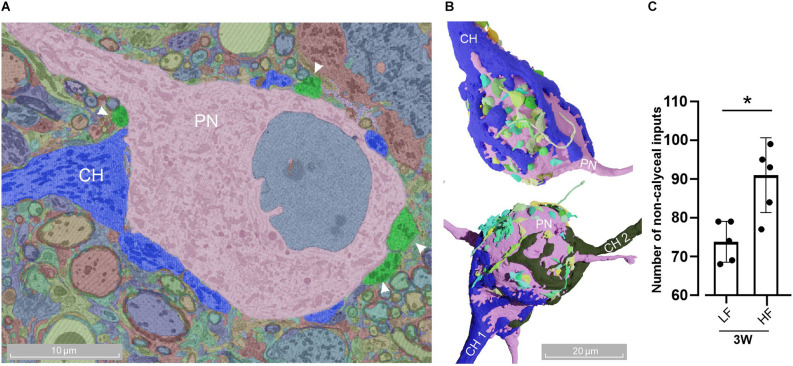
Adult MNTB cells receive multiple contacts in addition to calyx of Held inputs. **(A)** Representative micrograph of a MNTB cell (pink) receiving a calyx of Held (blue) and other non-calyceal inputs (green, arrowheads). **(B)** Three-dimensional reconstruction of a mono- (top) and poly-innervated (bottom) cell with non-calyceal afferences. The color code for non-calyceal inputs was automatically assigned by the platform webKnossos, without any particular connectivity significance. **(C)** The number of non-calyceal calyx inputs at 3 W displayed larger afferent contacts in the HF (90 ± 9, *n* = 5) than LF region (74 ± 5, *n* = 5, Wilcoxon, *p* = 0.028). Error bars indicate mean ± SEM. *p < 0.05.

**Figure 6:**
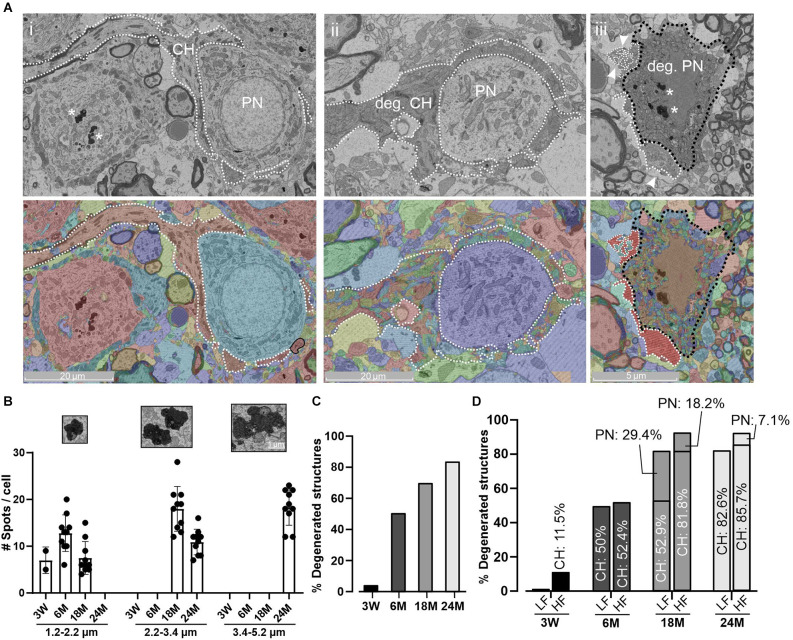
Age-related degeneration of PN and CH. **(A)** Representative micrograph (top) of a normal PN and CH structure at 18 M **(i)** and its corresponding segmentation (bottom). CH and PN are delimited with a white dashed or a black dashed line, respectively. Degeneration is evidenced in both structures at 18 M **(ii, iii)**. Note that the automatic detection failed to detect homogeneous structures evidenced by a mix of colors. Arrowheads indicate a fragmented CH terminal. Aging pigments are seen within the PNs (asterisks). **(B)** Quantification of age-pigments per cell. The size of aging spots was split in three ranges (1.2–2.2; 2.2–3.4, 3.4–5.2 μm) and grouped by age. Smaller spots were usually found at early stages with a progressive increase to higher size at 24 M. Error bars indicate mean ± SEM. **Inset**. Zoom-in of representative aging spots for each range. **(C)** Percentage of degenerated structures at different ages. In this case, both CH and PN structures were grouped. **(D)** Percentage of degenerated structures denoting the contribution of CH and PN by region and age. Note that PN degeneration appeared later than CH alterations and the HF region tends to be more affected.

### Manual volume annotation

To identify objects of interest, the segmented datasets were analyzed using the webKnossos open-source visualization and annotation software (Boergens et al., 2017). The structures of interest were principally identified based on the following criteria: PN between 20 and 30 μm in diameter (contacted at some point by at least one or more calyces); CH with synaptic boutons or innervations (recognizable by the presence of mitochondria, synaptic vesicles and active zones and axonal cone, see Figure 1). The calyx structure was confirmed after curation by its 3D reconstruction for 3 W, 6 M, and 18 M. In some cells with a high degeneration degree at 24 M, it was not easy to distinguish the PN from other cellular types. Thus, the evidence of an axonal cone in those calyces contacting a PN was needed to confirm the presence of a PN. For Figures 3–6, 12 cells were selected for each age to linearly represent the tonotopic (medio-lateral) distribution of the MNTB. Each segmented structure was associated with an identification number (ID) for posterior identification.

### Calyx and MNTB neuron reconstruction

With the segmentation as a starting point, the webKnossos merger mode was employed to create final volume annotation, as described in Boergens et al. (2017). Briefly, the initial segmentation had been biased towards split errors, resulting in a proto-segmentation that had no mergers in the structures identified. Then, a second-order agglomerator was used to reduce the number of splits. Lastly, segments were manually identified and marked by skeleton nodes that together created a complete volume model of the structure.

High-definition surface models were generated from data downloaded from webKnossos (Figures 3–5). These downloaded surface models (as STL files) were imported as volumetric reconstructions into Blender 3.2 (Stichting Blender Foundation, Amsterdam^3^). Calyx complexity was divided into three classes based on the number of swellings according to Grande and Wang (2011): 0–7 (Type 1), 8–14 (Type 2), and more than 15 swellings (Type 3). Since calyx type can be determined without segmentation, calyx reconstruction at 24 M for Figure 4Bv was made manually.

### Soma coverage measurement of calyceal terminals

To measure the percentage of the soma surface that is covered by calyceal terminals, the raw soma volume as annotated above was loaded into Python and dilated with a ball element of three pixels radius. This dilated volume was subtracted from the original soma volume, thereby creating a thin S shell. Then, the raw axon volume was loaded into Python and dilated with a ball element of six pixels radius. The output of this operation was called A. Then we created a manual soma annotation. To conserve annotation time, small errors in the soma outline (<20 px) were considered acceptable. This volume was also loaded. To make sure that this annotation always contained the soma surface it was dilated by a ball element of radius 30 pixels and named M. At this point, the thin S shell still contained trunks of dendrites to the extent that they were part of the supervoxels of the segmentation. Therefore, a new volume S’ was created that contained all voxels that were part of M and S. The soma coverage fraction was determined by summing up all voxels contained both in S’ and A and dividing that by the number of voxels contained in S’.

### MNTB along the tonotopic axis

Based on the automatic segmentation and posterior manual curation, PNs and CHs were identified. For Figures 2 and 3, the total number of cells in the whole stack for each age was taken into account for total density analysis. In the case of neuron density by tonotopic regions, we divided the whole tonotopic axis into three regions (medial, central, and lateral). After identification of the lateral and medial regions, the same cube (XYZ: 70 μm × 70 μm × 70 μm; 0.000343 mm^3^) was used for all databases and regions. The cube size was chosen in agreement with the following criteria: (a) to cover at least 3 cells in the tonotopic axis; (b) include at least 10 cells within the volume; and (c) not bigger than one-third of the tonotopic axis. In this case, we randomly placed these cubes four times in each region, and the number of cells was averaged. A cell is considered inside the cube when minimally 60% of its area is inside in at least two of the three planes. For Figures 3–6; 12 random cells along the tonotopic axes were chosen for quantification. Poly-innervated MNTB neurons were evaluated in each dataset (Figure 3B). For low and high-frequency comparisons, only those cells located in these regions were used to calculate the percentage of poly-innervated cells per region (Figures 2D, 3C).

#### Age-related synapse degeneration

A structure (CH or PN) was considered degenerated when non-uniform cellular or nuclear membranes were observed (Casey and Feldman, 1985). It should be noted that the degree of degeneration coincided with a decreased performance of the segmentation algorithm.

#### Statistics

All statistical tests were performed using a Python module for statistics (scipy.stats, version1.5.2). The group comparisons were done using Kruskal-Wallis and Wilcoxon rank-sum for comparisons between the two groups. For comparing the percentage of neuronal density by region (Figure 2D) and the poly-innervation by region (Figure 3C), we consider each age as independent of each other (*n* = 4). When multiple comparisons after the Kruskal-Wallis test were needed, the Bonferroni correction was applied to reduce false positives. Thus, the alpha value chosen for the Wilcoxon rank-sum test (*α* = 0.05) was divided by the number of multiple comparisons (in our case 3 due to comparison between: 3 W vs. 6 M, 3 W vs. 18 M, and 6 M vs. 18 M) reaching a new alpha value of *α* = 0.0167. *P*-values obtained by the Wilcoxon test were lower than this new α-value taking into account statistical differences in Figure 4B. All error bars indicate mean ± SEM.

## Results

### Auditory brainstem response along the life span

To examine whether auditory perception declines during the life span, ABRs were measured in mice at 3 W, 6, 18, and 24 M. Young mice (3 W) displayed normal (Zhang et al., 2022) and low ABR thresholds at all frequencies tested (Figure 1A). However, at 6 M the ABR thresholds were elevated at high frequencies and this progressed toward the lower frequency regions in older animals. While at 18 M only middle frequencies were conserved, a profound hearing loss was seen at 24 M. These results agree with previous ones describing the initial high-frequency decline at 3 months in C57BL/6J mice line (Zhang et al., 2022).

### Tonotopic distribution of MNTB cells

In order to study the impact of diverse grades of HL at the brainstem level, we decided to study morphological alterations in the MNTB (Figure 1B) at different ages by SBEM (Figures 1C,D). This technique permits big volume data analysis enabling structural studies along the entire MNTB mediolateral axis with a high spatial resolution required for a comprehensive ultrastructure analysis. The analysis was performed in mice of different ages (3 W, 6, 18, and 24 M), resulting in 3D-aligned volumes of (XYZ) 122.5 μm × 541.8 μm × 92.0 μm, 136.1 μm × 560.8 μm × 77.2 μm, 125.6 μm × 548.9 μm × 76.0 μm, 152.9 μm × 583.1 μm × 92.4 μm at 15 nm × 15 nm × 50 nm voxel size for 3 W, 6, 18, and 24 M, respectively (Figure 1D). Quantification was done by machine learning-based image processing tools (segmentation, see Section “Materials and methods”) and a subsequent manual curation using webKnossos, an open-source visualization and annotation software (Boergens et al., 2017).

We quantified the number of cells in the whole MNTB at different ages (Figures 2A,B). An overall decrease in cell counts was observed with age (3 W: 221 cells; 6 M: 172 cells; 18 M: 155 cells; 24 M: 165 cells). Due to the fact that block size was not the same for all databases (3 W: 0.0036 mm^3^; 6 M: 0.0038 mm^3^; 18 M: 0.0041 mm^3^; 24 M: 0.0051 mm^3^), we normalized the number of cells to the volume analyzed. Using this metric, the cell density shrank monotonically from 3 W to 24 M (3 W: 61,146; 6 M: 44,925; 18 M: 38,396; 24 M: 32,114 cells/mm^3^, Figure 2C). The global neuron loss was largest from 3 W to 6 M (27%), compared to a loss of 15% between 6 M and 18 M and 16% between 18 M and 24 M. Despite the parity observed at 24 M (LF: 47.9 ± 2.7 vs. HF: 52.1 ± 2.7, *n* = 4, Wilcoxon, *p* = 0.39), cell density was lower in the high frequency region at 3 W (LF: 55.7 ± 1.5 vs. HF: 44.3 ± 1.5, *n* = 4, Wilcoxon, *p* = 0.021) and 6 M (LF: 55.7 ± 1.2 vs. HF: 44.3 ± 1.2, *n* = 4, Wilcoxon, *p* = 0.031), being more evident at 18 M (LF: 61.6 ± 3.2 vs. HF: 38.4 ± 3.2, *n* = 4, Wilcoxon, *p* = 0.043; Figure 2D), in line with a relatively higher ABR threshold in the high-frequency region as from 6 M onwards (Figure 1A).

The EM block contained just a part of the MNTB. Based on our database (Figures 1D, 2B), the medio-lateral (tonotopic) and the dorso-vental axes were better represented than the rostro-caudal (Z) axis (80 μm depth). While the average depth of the MNTB is about 800 μm (Sonntag et al., 2009), only a tenth of the MNTB in the rostro-caudal axis was sampled in our datasets. Considering the cell count in our analysis in a young, but mature animal (3 W: 221 PN), we can predict about 2,200 PN per MNTB, a number of cells in agreement with a previous report at a young age (Krohs et al., 2021). This estimation highlights that the age-related decline in the number of cells is associated with the aging process instead of a technical or analytical problem.

### Poly-innervation of MNTB cells by calyceal structures

It has been extensively described that presynaptic CH terminals make multiple small contacts on MNTB neurons during the early developmental critical period (first postnatal week), followed by an early stage of functional and structural transformation (Kandler and Friauf, 1993; Taschenberger et al., 2002; Wimmer et al., 2006; Rodríguez-Contreras et al., 2008). During this pruning, multiple inputs strengthen and compete until a final single innervation is established (Holcomb et al., 2013; Sierksma et al., 2020). Recently, it was reported that the MNTB can still display multiple CH inputs in young mice even after the critical period (3 W and 4 W; Milinkeviciute et al., 2019, 2021). To study if poly-innervation is also present at mature ages, we next examined the proportion of MNTB neurons receiving more than one calyceal presynaptic terminal in older animals (Figure 3A). A complete reconstruction of the datasets showed an average of approximately 10% of poly-innervated MNTB neurons at all ages [poly-innervated/total number of cells; 3 W: 20/163 (12%), 6 M: 17/118 (14%), 18 M: 11/126 (9%); 24 M: 12/107 (11%), Figure 3B]. Despite the similar ratio observed at 24 M comparing LF 2/4 [50%] vs. HF 2/4 [50%], it is important to note that the large percentage of poly-innervated MNTB neurons was higher in the low compared to the high frequency region at 3 W [LF 4/6 (67%) vs. HF 2/6 (33%)], 6 M [LF 6/8 (75%) vs. HF 2/8 (25%)], and 18 M [LF 3/4 (75%) vs. HF 1/4 (25%)]; *n* = 4, Wilcoxon, *p* = 0.03; Figure 3C). Taken together, these results demonstrate that poly-innervation of MNTB neurons is conserved beyond the critical period.

### Coverage area of MNTB PN cells by the CH

During the first two postnatal weeks, the CH structure develops from a single cup-shaped to a more complex fenestrated structure (Kandler and Friauf, 1993; Rowland et al., 2000; Wimmer et al., 2006). According to reported data (Ford et al., 2009; Grande and Wang, 2011; Grande et al., 2014) and that presented in this work (Figure 4A), there are at least three types of morphological mature CHs phenotypes, which lead to different coverage areas of MNTB PN cells. Similar to that described by Grande and Wang (2011), we did not observe any tonotopic distribution of calyx complexity along the tonotopic axis. We chose 12 random PNs along the tonotopic axes (Figures 4B,C) to compare several morphological aspects at different ages. The area of the PNs was similar for 3 W (1,598 ± 43 μm^2^) and 6 M (1,783 ± 115 μm^2^, *n* = 12, Wilcoxon, *p* = 0.17), but was reduced at 18 M (1,389 ± 45 μm^2^, *n* = 12, Wilcoxon, *p* = 0.005, Figure 4Bi). PNs volume differences were observed between 3 W and 6 M (3 W: 3,743 ± 120 μm^3^, 6 M: 5,396 ± 457 μm^3^; *n* = 12; *p* = 0.002) and between 6 M and 18 M (18 M: 3,545 ± 153 μm^3^; *n* = 12, Wilcoxon, *p* = 0.001, Figure 4Bii). Comparable mean volumes of CH terminals were observed at all ages: 3 W (437 ± 49 μm^3^), 6 M (532 ± 65 μm^3^), and 18 M (490 ± 33 μm^3^; *n* = 12, Kruskal-Wallis, *p* = 0.92, Figure 4Biii). Interestingly, a reduction in CHs volume dispersion was observed at 18 M (CV: 0.23) compared to other ages (CV 3 W: 0.43; CV 6 M: 0.37; Figure 4Biii). This was mainly due to the lack of low volume CHs (Figure 4Biii). Complex calyces (Type 3, boxes) displayed larger volumes (Figure 4Biii) than simple ones (Type 1, circles).

A relevant morphological feature with physiological implications is the area of a PN covered by the CH. PNs were similarly covered by the calyx at all ages analyzed (3 W: 31.9 ± 3.7%, CV: 0.45, *n* = 15; 6 M: 33.8 ± 3.6%, CV: 0.41, *n* = 15, and 18 M: 43.3 ± 2.5%, CV: 0.19, *n* = 12, Kruskal-Wallis, *p* = 0.12, Figure 4Biv). However, a reduced data dispersion was observed at 18 M, in line with more complex structures at old ages (Figure 4Bv, Chi-squared, *p* = 0.0016).

In order to analyze the tonotopic distribution of PN coverage for each age we compared the properties of four randomly selected cells in the high-frequency region to four randomly selected cells in the low-frequency region. The 3 W and 6 M sample sets contained both single and poly-innervated neurons. Since no correlation was found when single calyces were considered (Figure 4C, top panel for 3 W and 6 M), we decided to sum up the coverage areas of those calyces that innervated the same PN (poly-innervated PNs). At 3 W there was a linear correlation between the total coverage area and the tonotopic axes (*R*^2^ = 0.93), with higher values at low (45.4 ± 2.9%) compared to high-frequency areas (28.1 ± 1.7%; *n* = 4 LF and *n* = 4 HF neurons, Wilcoxon, *p* = 0.02; Figure 4C). This correlation was absent at 6 M (*R*^2^ = 0.032, *n* = 4 LF and *n* = 4 HF neurons, Wilcoxon, *p* = 0.77) and at 18 M (*R*^2^ = 0.045; *n* = 4 LF and *n* = 4 HF neurons, Wilcoxon, *p* = 1). Additionally, at both 6 and 18 M the covered area was close to 40% (6 M: LF = 42.9 ± 2.3%, HF: 42.1 ± 4.1; 18 M: LF = 44.7 ± 7.2%, HF: 41.6 ± 1.2), similar to that observed in the low-frequency region in 3 W old mice. Taken together, these results suggest a prevalence of complex calyx structures in older animals and a disruption of the coverage pattern along the tonotopic axis in an age-dependent manner.

### MNTB cells receive multiple contacts in addition to calyx inputs in aged animals

It has been extensively described that the calyx of Held is the most prominent innervation onto the PNs of the MNTB. However, they also receive somatic inhibitory (Awatramani et al., 2004) and also excitatory inputs (Guinan and Li, 1990; Hamann et al., 2003; Hoffpauir et al., 2006; Rodríguez-Contreras et al., 2008), which exert a modulatory function (Zhang et al., 2021). This large body of reports describes these non-calyceal innervations in prehearing rodents. Figures 5A,B show the presence of different types of inputs onto MNTB neurons. While the presence of these inputs was observed at all ages (data not shown), we analyzed its prevalence at the hearing-functionally-intact 3 W old mice (Figure 1A). We selected five representative neurons per each region and quantified the number of non-calyceal inputs onto the PN (Figure 5B). We found that PNs in the low-frequency region received a lower average number 74 ± 5 (*n* = 5; CV = 0.07) of non-calyceal inputs compared to the high frequency region 90 ± 9 (*n* = 5; CV = 0.1; Wilcoxon, *p* = 0.028). Note that MNTB neurons contacted by two CH (poly-innervated), were also contacted by other afferents (Figure 5B, lower panel), suggesting that a modulatory effect is also present in these cells.

### Age-related degeneration

For the morphological analysis shown in Figures 1–5; only cells with a conserved cytoarchitecture on both synaptic components (the CH and the PN) were taken into account. However, morphological alterations have been described in older animals (Casey and Feldman, 1985). The present electron microscopy analysis evidenced several morphological phenotypes associated with cellular deterioration, including a non-uniform cellular and/or nuclear membrane. Since small electrodense spots can be related to methodological artifacts, we focused our analysis on the largest spots (more than 1.2 μm) which are frequently associated with metabolic deficits of proteins, sugars, or lipids (Moreno-García et al., 2018). Electrodense pigments (Figure 6A) usually dispersed within the cytoplasm were detected in MNTB cells even in 3 W-old mice. At 3 W, the majority of these spots were less than 1 μm in diameter and just a few larger spots (between 1.2 and 2.2 μm) were occasionally observed (Figure 6B). However, the incidence of these spots increased with age, reaching its maximal prevalence and size at 24 M (Figure 6B).

Another age-related morphological observation was the presence of degenerated CH terminals and PN (Figures 6Aii,iii) which increased with age (Figure 6C, left panel). Interestingly, CH disintegration preceded PN degeneration, being more pronounced within the high-frequency region (Figure 6C, right panel; Chi-squared, *p* = 0.035). It should be noted that at 18 M and at 24 M, the presence of degenerated PNs with intact CH was observed (18 M: 11 degenerated PNs with nine normal CHs; 24 M: four degenerated PNs with two normal CHs; data not shown). Taken together, these data are in line with a premature decline of auditory high-frequency perception.

## Discussion

In the present work, we used high-throughput volume EM and artificial intelligence assisted image processing techniques to characterize age-related structural changes in a central auditory nucleus. Despite recent developments in volume EM techniques, it is still technically challenging and resource-demanding to acquire high-resolution serial-sectioning datasets in the nanoliter volume range. In addition, the segmentation and manual curation turn out to be time-consuming for such large datasets, even with an established analysis pipeline. However, the ability of 3D electron microscopy to image tissue in an unbiased fashion (Kornfeld et al., 2017; Schmidt et al., 2017; Svara et al., 2018; Motta et al., 2019; Karimi et al., 2020; Morgan and Lichtman, 2020; Hua et al., 2021) could thereby generate unexpected results and trigger further studies using, for example, X-ray computed tomography (Bosch et al., 2022). Nevertheless, our work complements prior 3D EM studies [P2, P3, P4, P6, and P9 (Holcomb et al., 2013); P7, P21 (Thomas et al., 2019); P21, P180, P540, and P720, this study] providing a framework for studying connectomic and morphomic changes in the MNTB over the lifespan of rodents. To the best of our knowledge, previous EM works on the MNTB primarily focused on pre-hearing states (Hoffpauir et al., 2006; Holcomb et al., 2013) or on very young animals (Sinclair et al., 2017; Thomas et al., 2019). However, the hallmarks of aging remained largely elusive, except for the limited insights obtained from a single work on older rats (Casey and Feldman, 1985), due to the small tissue block analyzed in that study. In the present manuscript, we covered the whole mediolateral (tonotopic) MNTB axis from very young post-hearing to old mice. Overall, our data shows MNTB age-related morphological alterations leading to brainstem circuit remodeling.

### Animal model of ARHL

C57BL/6J is the most widely used inbred strain in which many transgenic mice lines have been produced (Bryant, 2011) and it is commonly used in brain slice recordings. This model has also been extensively studied because of the presence of a type of age-related hearing loss (Henry and Lepkowski, 1978; Willott, 1986; Li et al., 1993; Taberner and Liberman, 2005). Unlike other strains, C57BL/6J displays accelerated hearing loss (Mikaelian et al., 1974; Henry and Lepkowski, 1978; Mikaelian, 1979), making this genetic background a problematic model for many auditory physiology studies. However, it is a suitable model for studying age-related degenerative problems due to a progressive sensorineural impairment, occurring primarily after the auditory system has matured (Willott, 1986). Moreover, the early degradation of high frequency hearing, encourages a comparative study between low and high-frequency tonotopic regions along the life span. The observed normal auditory profile at 3 W with an initial hearing decline at 6 M initially affecting the high frequencies, is in agreement with that previously reported (Mikaelian, 1979; Spongr et al., 1997; Sonntag et al., 2009; Zhang et al., 2022). Since the C57BL/6J mice represent a very specific model with early onset of age-related hearing loss, one might expect different results from other mouse models showing minor age-related hearing loss.

### Age-related decline in MNTB neuron density

Quantitative comparison of the MNTB datasets displayed an age-related decline in the density of MNTB neuron, where high frequencies at 3 W, 6 M, and 18 M were more affected than low frequencies, in line with the ABRs. One possible scenario for the lack of tonotopic differences at 24 M might be the presence of both, hearing loss and aging occurring simultaneously. Thus, the age-related deterioration of synaptic transmission (partially attributed to mitochondrial impairments in elderly mice, Singh et al., 2018), can contribute to neuronal cell loss due to the hearing deficit seen in young animals. However, the decline of total cell density with aging is in accordance with a progressive emergence of age-related degeneration phenotypes of CHs and PNs, which include the presence of age pigments and degenerated structures. While the molecular underpinnings of age-related brain changes are still not well understood (Moreno-García et al., 2018), the identity of age pigments can be associated with lipofuscin (Casey and Feldman, 1982, 1985), defined as electron-dense pigment granules that accumulate progressively over time in lysosomes of postmitotic cells (Terman and Brunk, 1998). In addition, the buildup of metabolic anomalies on lipids, metals, and proteins can also promote the formation of these precipitates (Moreno-García et al., 2018). Note that age pigments were detected earlier than degenerated structures, suggesting that these precipitates could be involved in the deteriorating process. Calyx of Held disintegration preceded the degeneration of PNs and it was much more pronounced within the high-frequency region. Taking into account that 6 M is generally considered a young age for mice, but that the C57BL/6J mouse strain displays a reduced auditory input, one can speculate that the morphological alterations at this age are more related to deafferentation, rather than an age-related decline. Furthermore, the early appearance of CH alterations at 6 M without PN degeneration, further indicates that, at this early age, alterations are due to a lack of auditory input. However, the fact that at 18 M degenerated PNs were observed in the presence of intact CH, indicates intrinsic age-related MNTB morphological alterations in addition to those resulting from deafferentation.

It is worth noting that at 3 W the number of non-calyceal inputs displayed a medio-lateral gradient, when compared to the medio-lateral gradient of the coverage area of PNs by CHs. The nature of the non-calyceal modulatory neurotransmission engages both inhibitory and excitatory neurotransmitters. Thus, PNs of the MNTB receive glycinergic and/or GABAergic inhibitory inputs from the ipsilateral ventral nucleus of the trapezoid body (VNTB) and the MNTB itself (Kuwabara et al., 1991; Albrecht et al., 2014; Dondzillo et al., 2016) and excitatory cholinergic input from the superior olivary nuclei (Zhang et al., 2021). Since a higher expression level of GAD-67 (McCullagh et al., 2017), GABA vesicular transporter (VGAT; Yu and Wang, 2022), and glycine transporter 2 (GlyT2; McCullagh et al., 2017) is observed in the lateral side of the MNTB, one can propose that the increased number of non-calyceal inputs in the medial region could arise from the cholinergic innervation. However, based on the lack of sufficient knowledge, an increase of other neuromodulatory inputs cannot be precluded.

### Poly-innervation of MNTB cells is sustained beyond the critical period

During development, MNTB neurons receive multiple proto-calyceal synaptic inputs from the globular bushy cells located in the cochlear nucleus. Already before hearing onset (around P12–P14), just one giant calyx terminal contacts one postsynaptic cell, after a process of elimination of multiple small inputs, pruning and strengthening of the “winner” terminal (Kuwabara et al., 1991; Hoffpauir et al., 2006; Holcomb et al., 2013; Sierksma et al., 2020). In some pathological conditions like the deletion of bone morphogenetic protein (Xiao et al., 2013), alteration in the microglia (Milinkeviciute et al., 2019, 2021) or potentiation of the medial olivocochlear system (Di Guilmi et al., 2019), the presence of multiple calyces on a principal MNTB has been reported beyond the critical period. In addition, it has recently been reported that in young wild type mice (3–4 postnatal weeks) a subset of MNTB neurons has multiple calyceal inputs (Milinkeviciute et al., 2019, 2021). Notably, our work demonstrates that an average of approximately 10% of poly-innervated MNTB cells is conserved along the mouse lifespan and its prevalence is larger in the lateral part of the MNTB, a region where low frequencies are processed. Calyx of Held fenestration can be considered a maturity marker and those CH located in the high-frequency region fenestrate earlier than those located in the low-frequency region (Ford et al., 2009). This temporary developmental gradient along the tonotopic axis is established during the critical period before the hearing onset (P12–P14). Considering that calyceal competition occurs during the same developmental windows (Rodríguez-Contreras et al., 2008; Sierksma et al., 2020) and that the maturation of neurotransmission progresses from high to low frequencies along the tonotopic axis (Yu and Wang, 2022), one can speculate that the only plastic window for eliminating terminals is during the critical period. Since low frequencies mature later, one can consider that not all MNTB cells located in this region mature before the end of the critical period, leaving an incomplete elimination of its calyceal inputs. However, this developmental reminiscence hypothesis does not preclude a potential functional relevance of these poly-innervated MNTB cells in processing auditory information in the mature circuit as detailed in the next section. Thus, future studies are needed to determine the physiological role of poly-innervated mature PNs.

### Correct encoding of low frequencies may be essential for long-distance communication

Neurons that encode low-frequency signals exhibit phase locking (Joris and Trussell, 2018; Rutherford et al., 2021), which plays a fundamental role in generating an output in phase with the stimulus frequency. In a natural environment, low-frequency sounds travel greater distances than high-frequency sounds (Joris and Trussell, 2018; Capshaw et al., 2022). In particular, some rodents have been observed to vocalize with low-frequency sounds in order to communicate with each other when separated by long distances (Briggs and Kalcounis-Rueppell, 2011). We can speculate that the larger coverage area observed in the lateral low-frequency principal neurons (Figure 4C), even at the cost of more than one calyx being involved (Figure 3C), might enable the fidelity of synaptic transmission. Interestingly, especially in older animals, the predominant occurrence of complex calyx morphologies (Type 3), would help maintain synaptic activity along the lifespan. This type of complex calyx morphology displays fewer postsynaptic failures and lower short-term depression than simple ones at comparable frequencies (Grande and Wang, 2011).

### Age-related sound localization deficits

Many brainstem auditory structures are involved in sound localization (Masterton et al., 1967). Particularly, the MNTB plays a role in the localization of auditory stimuli by relaying inputs from the contralateral ventral cochlear nucleus to the ipsilateral medial superior olive (Borst and Soria van Hoeve, 2012), which is involved in azimuthal localization for low-frequency sounds (Kulesza and Grothe, 2015; Cadenas et al., 2020). Aging is frequently accompanied by hearing loss, affecting sound source localization (Xiong et al., 2022) and it may impair spatial perception (Akeroyd, 2014). Older adults show reduced accuracy and precision in auditory localization tasks (Dobreva et al., 2011; Freigang et al., 2015), possibly due to deficits in processing interaural temporal differences, interaural level differences, and monaural spectral cues (Gallun, 2021). Future work should focus on how age-related changes, if occurring differently between ears, impact the binaural function. Taking into account the relay role of the MNTB on brainstem auditory structures involved in sound localization, the age-dependent degenerative process described in the present manuscript (increasing age pigments, calyx, and PN degeneration) probably contributes to the reported age-related decline in auditory function.

## Data Availability Statement

The raw data supporting the conclusions of this article will be made available by the authors, without undue reservation.

## Ethics Statement

The animal study was reviewed and approved by ethical committee of Shanghai Ninth People’s Hospital (No. SH9H-2020-A420-1).

## Author Contributions

DC: analysis, manual curation, and 3D reconstruction. YH: study design, writing, and funding. HW: analysis. WH: sample preparation and data acquisition. FW: data acquisition. ABE: supervise, writing, and funding. KMB: supervise, analysis, and writing. MNDG: supervise, study design, and writing. All authors contributed to the article and approved the submitted version.

## Footnotes

^1^ https://www.who.int/news-room/fact-sheets/detail/deafness-and-hearing-loss

^2^ https://arxiv.org/abs/1706.00120

^3^ http://www.blender.org

## References

[B1] Akeroyd M. A. (2014). An overview of the major phenomena of the localization of sound sources by normal-hearing, hearing-impaired and aided listeners. Trends Hear. 18, 10–16. 10.1177/2331216514560442 25492094 PMC4271773

[B2] Albrecht M. Henke J. Tacke S. Markert M. Guth B. (2014). Effects of isoflurane, ketamine-xylazine and a combination of medetomidine, midazolam and fentanyl on physiological variables continuously measured by telemetry in Wistar rats. BMC Vet. Res. 10:198. 10.1186/s12917-014-0198-3 25149627 PMC4363998

[B3] Awatramani G. B. Turecek R. Trussell L. O. (2004). Inhibitory control at a synaptic relay. J. Neurosci. 24, 2643–2647. 10.1523/JNEUROSCI.5144-03.2004 15028756 PMC6729505

[B4] Baydyuk M. Xu J. Wu L. G. (2016). The calyx of held in the auditory system: structure, function and development. Hear. Res. 338, 22–31. 10.1016/j.heares.2016.03.009 27018297 PMC4967386

[B5] Bock D. D. Lee W.-C. A. Kerlin A. M. Andermann M. L. Hood G. Wetzel A. W.. (2011). Network anatomy and *in vivo* physiology of visual cortical neurons. Nature 471, 177–182. 10.1038/nature09802 21390124 PMC3095821

[B6] Boergens K. M. Berning M. Bocklisch T. Bräunlein D. Drawitsch F. Frohnhofen J.. (2017). WebKnossos: efficient online 3D data annotation for connectomics. Nat. Methods 14, 691–694. 10.1038/nmeth.4331 28604722

[B7] Boero L. E. Castagna V. C. Terreros G. Moglie M. J. Silva S. Maass J. C.. (2020). Preventing presbycusis in mice with enhanced medial olivocochlear feedback. Proc. Natl. Acad. Sci. U S A 117, 11811–11819. 10.1073/pnas.2000760117 32393641 PMC7261056

[B8] Borst J. G. G. Soria van Hoeve J. (2012). The calyx of held synapse: from model synapse to auditory relay. Annu. Rev. Physiol. 74, 199–224. 10.1146/annurev-physiol-020911-153236 22035348

[B9] Bosch C. Ackels T. Pacureanu A. Zhang Y. Peddie C. J. Berning M.. (2022). Functional and multiscale 3D structural investigation of brain tissue through correlative *in vivo* physiology, synchrotron microtomography and volume electron microscopy. Nat. Commun. 13:2923. 10.1038/s41467-022-30199-6 35614048 PMC9132960

[B10] Briggman K. L. Helmstaedter M. Denk W. (2011). Wiring specificity in the direction-selectivity circuit of the retina. Nature 471, 183–188. 10.1038/nature09818 21390125

[B11] Briggs J. R. Kalcounis-Rueppell M. C. (2011). Similar acoustic structure and behavioural context of vocalizations produced by male and female California mice in the wild. Anim. Behav. 82, 1263–1273. 10.1016/j.anbehav.2011.09.003

[B12] Bryant C. D. (2011). The blessings and curses of C57BL/6 substrains in mouse genetic studies. Ann. N Y Acad. Sci. 1245, 31–33. 10.1111/j.1749-6632.2011.06325.x 22211972 PMC4944652

[B13] Burke K. Screven L. A. Kobrina A. Charlton P. E. Schrode K. Villavisanis D. F.. (2022). Effects of noise exposure and aging on behavioral tone detection in quiet and noise by mice. eNeuro 9:ENEURO.0391–21.2022. 10.1523/ENEURO.0391-21.2022 35613853 PMC9191765

[B14] Cadenas L. T. Fischl M. J. Weisz C. J. C. (2020). Synaptic inhibition of medial olivocochlear efferent neurons by neurons of the medial nucleus of the trapezoid body. J. Neurosci. 40, 509–525. 10.1523/JNEUROSCI.1288-19.2019 31719165 PMC6961997

[B15] Capshaw G. Vicencio-Jimenez S. Screven L. A. Burke K. Weinberg M. M. Lauer A. M. (2022). Physiological evidence for delayed age-related hearing loss in two long-lived rodent species (*Peromyscus leucopus* and *P. californicus*). JARO J. Assoc. Res. Otolaryngol. 23, 617–631. 10.1007/s10162-022-00860-4. 35882705 PMC9613845

[B16] Casey M. A. Feldman M. L. (1982). Aging in the rat medial nucleus of the trapezoid body I. Light microscopy. Neurobiol. Aging 3, 187–195. 10.1016/0197-4580(82)90039-2 6298647

[B17] Casey M. A. Feldman M. L. (1985). Aging in the rat medial nucleus of the trapezoid body. II. Electron microscopy. J. Comp. Neurol. 232, 401–413. 10.1002/cne.902320311 3973099

[B18] Caspary D. M. Ling L. Turner J. G. Hughes L. F. (2008). Inhibitory neurotransmission, plasticity and aging in the mammalian central auditory system. J. Exp. Biol. 211, 1781–1791. 10.1242/jeb.013581 18490394 PMC2409121

[B19] Collins J. G. (1997). Prevalence of selected chronic conditions: United States, 1990–1992. Vital Health Stat. 10, 1–89. 9046223

[B20] Deal J. A. Goman A. M. Albert M. S. Arnold M. L. Burgard S. Chisolm T.. (2018). Hearing treatment for reducing cognitive decline: design and methods of the aging and cognitive health evaluation in elders randomized controlled trial. Alzheimer’s Dement. Transl. Res. Clin. Interv. 4, 499–507. 10.1016/j.trci.2018.08.007 30364572 PMC6197326

[B21] Di Guilmi M. N. Boero L. E. Castagna V. C. Rodríguez-Contreras A. Wedemeyer C. Gómez-Casati M. E.. (2019). Strengthening of the efferent olivocochlear system leads to synaptic dysfunction and tonotopy disruption of a central auditory nucleus. J. Neurosci. 39, 7037–7048. 10.1523/JNEUROSCI.2536-18.2019 31217330 PMC6733545

[B22] Dobreva M. S. O’Neill W. E. Paige G. D. (2011). Influence of aging on human sound localization. J. Neurophysiol. 105, 2471–2486. 10.1152/jn.00951.2010 21368004 PMC3094163

[B23] Dondzillo A. Thompson J. A. Klug A. (2016). Recurrent inhibition to the medial nucleus of the trapezoid body in the mongolian gerbil (*Meriones unguiculatus*). PLoS One 11, e0160241. 10.1371/journal.pone.0160241 27489949 PMC4973988

[B24] Elgoyhen A. B. (2022). The α9α10 nicotinic acetylcholine receptor: a compelling drug target for hearing loss? Expert Opin. Ther. Targets 26, 291–302. 10.1080/14728222.2022.2047931 35225139 PMC9007918

[B25] Ford M. C. Grothe B. Klug A. (2009). Fenestration of the calyx of Held occurs sequentially along the tonotopic axis, is influenced by afferent activity and facilitates glutamate clearance. J. Comp. Neurol. 514, 92–106. 10.1002/cne.21998 19260071

[B26] Freigang C. Richter N. Rübsamen R. Ludwig A. A. (2015). Age-related changes in sound localisation ability. Cell Tissue Res. 361, 371–386. 10.1007/s00441-015-2230-8 26077928

[B27] Frisina R. D. Walton J. P. (2006). Age-related structural and functional changes in the cochlear nucleus. Hear. Res. 216–217, 216–223. 10.1016/j.heares.2006.02.003 16597491

[B28] Fu B. Le Prell C. Simmons D. Lei D. Schrader A. Chen A. B.. (2010). Age-related synaptic loss of the medial olivocochlear efferent innervation. Mol. Neurodegener. 5:53. 10.1186/1750-1326-5-53 21110869 PMC3000387

[B29] Funke J. Tschopp F. Grisaitis W. Sheridan A. Singh C. Saalfeld S.. (2019). Large scale image segmentation with structured loss based deep learning for connectome reconstruction. IEEE Trans. Pattern Anal. Mach. Intell. 41, 1669–1680. 10.1109/TPAMI.2018.2835450 29993708

[B30] Gallun F. J. (2021). Impaired binaural hearing in adults: a selected review of the literature. Front. Neurosci. 15, 1–22. 10.3389/fnins.2021.610957 33815037 PMC8017161

[B31] Gates G. A. Mills J. H. (2005). Presbycusis. Lancet 366, 1111–1120. 10.1016/S0140-6736(05)67423-5 16182900

[B32] Gour A. Boergens K. M. Heike N. Hua Y. Laserstein P. Song K.. (2021). Postnatal connectomic development of inhibition in mouse barrel cortex. Science 371:eabb4534. 10.1126/science.abb4534 33273061

[B33] Grande G. Wang L.-Y. (2011). Morphological and functional continuum underlying heterogeneity in the spiking fidelity at the calyx of Held synapse *in vitro*. J. Neurosci. 31, 13386–13399. 10.1523/JNEUROSCI.0400-11.2011 21940432 PMC6623283

[B34] Grande G. Negandhi J. Harrison R. V. Wang L. Y. (2014). Remodelling at the calyx of Held-MNTB synapse in mice developing with unilateral conductive hearing loss. J. Physiol. 592, 1581–1600. 10.1113/jphysiol.2013.268839 24469075 PMC3979613

[B35] Guinan J. J. Li R. Y. S. (1990). Signal processing in brainstem auditory neurons which receive giant endings (calyces of Held) in the medial nucleus of the trapezoid body of the cat. Hear. Res. 49, 321–334. 10.1016/0378-5955(90)90111-2 2292504

[B36] Haile L. M. Kamenov K. Briant P. S. Orji A. U. Steinmetz J. D. Abdoli A.. (2021). Hearing loss prevalence and years lived with disability, 1990–2019: findings from the global burden of disease study 2019. Lancet 397, 996–1009. 10.1016/S0140-6736(21)00516-X 33714390 PMC7960691

[B37] Hamann M. Billups B. Forsythe I. D. (2003). Non-calyceal excitatory inputs mediate low fidelity synaptic transmission in rat auditory brainstem slices. Eur. J. Neurosci. 18, 2899–2902. 10.1111/j.1460-9568.2003.03017.x 14656340

[B38] Held H. (1893). Die centrale Gehörleitung. Arch. Anat. Physiol. Anat. Abt. 17, 201–248.

[B39] Helmchen F. Borst J. G. Sakmann B. (1997). Calcium dynamics associated with a single action potential in a CNS presynaptic terminal. Biophys. J. 72, 1458–1471. 10.1016/S0006-3495(97)78792-7 9138591 PMC1184528

[B40] Henry K. R. Lepkowski C. M. (1978). Evoked potential correlates of genetic progressive hearing loss: age-related changes from the ear to the inferior colliculus of c57BL/6 and CBA/j mice. Acta Otolaryngol. 86, 366–374. 10.3109/00016487809107515 716859

[B41] Hoffpauir B. K. Grimes J. L. Mathers P. H. Spirou G. A. (2006). Synaptogenesis of the calyx of held: rapid onset of function and one-to-one morphological innervation. J. Neurosci. 26, 5511–5523. 10.1523/JNEUROSCI.5525-05.2006 16707803 PMC6675295

[B42] Holcomb P. S. Hoffpauir B. K. Hoyson M. C. Jackson D. R. Deerinck T. J. Marrs G. S.. (2013). Synaptic inputs compete during rapid formation of the calyx of Held: a new model system for neural development. J. Neurosci. 33, 12954–12969. 10.1523/JNEUROSCI.1087-13.2013 23926251 PMC3735879

[B43] Hua Y. Ding X. Wang H. Wang F. Lu Y. Neef J.. (2021). Electron microscopic reconstruction of neural circuitry in the cochlea. Cell Rep. 34:108551. 10.1016/j.celrep.2020.108551 33406431

[B44] Hua Y. Laserstein P. Helmstaedter M. (2015). Large-volume en-bloc staining for electron microscopy-based connectomics. Nat. Commun. 6:7923. 10.1038/ncomms8923 26235643 PMC4532871

[B45] Hughes L. F. Turner J. G. Parrish J. L. Caspary D. M. (2010). Processing of broadband stimuli across A1 layers in young and aged rats. Hear. Res. 264, 79–85. 10.1016/j.heares.2009.09.005 19772906 PMC2868092

[B46] Jean-Baptiste M. Morest D. K. (1975). Transneuronal changes of synaptic endings and nuclear chromatin in the trapezoid body following cochlear ablations in cats. J. Comp. Neurol. 162, 111–133. 10.1002/cne.901620107

[B47] Joris P. X. Trussell L. O. (2018). The calyx of held: a hypothesis on the need for reliable timing in an intensity-difference encoder. Neuron 100, 534–549. 10.1016/j.neuron.2018.10.026 30408442 PMC6263157

[B48] Kandler K. Friauf E. (1993). Pre- and postnatal development of efferent connections of the cochlear nucleus in the rat. J. Comp. Neurol. 328, 161–184. 10.1002/cne.903280202 8423239

[B49] Karimi A. Odenthal J. Drawitsch F. Boergens K. M. Helmstaedter M. (2020). Cell-type specific innervation of cortical pyramidal cells at their apical dendrites. eLife 9:e46876. 10.7554/eLife.46876 32108571 PMC7297530

[B50] Kasthuri N. Hayworth K. J. Berger D. R. Schalek R. L. Conchello J. A. Knowles-Barley S.. (2015). Saturated reconstruction of a volume of neocortex. Cell 162, 648–661. 10.1016/j.cell.2015.06.054 26232230

[B51] Kornfeld J. Benezra S. E. Narayanan R. T. Svara F. Egger R. Oberlaender M.. (2017). EM connectomics reveals axonal target variation in a sequence-generating network. eLife 6:e24364. 10.7554/eLife.24364 28346140 PMC5400503

[B52] Krohs C. Körber C. Ebbers L. Altaf F. Hollje G. Hoppe S.. (2021). Loss of miR-183/96 alters synaptic strength via pre- and postsynaptic mechanisms at a central synapse. J. Neurosci. 41, 0139–0140. 10.1523/JNEUROSCI.0139-20.2021 34193555 PMC8360680

[B53] Kronander E. Clark C. Schneggenburger R. (2018). Role of BMP signaling for the formation of auditory brainstem nuclei and large auditory relay synapses. Dev. Neurobiol. 79:dneu.22661. 10.1002/dneu.22661 30548566

[B54] Kulesza R. J. Grothe B. (2015). Yes, there is a medial nucleus of the trapezoid body in humans. Front. Neuroanat. 9, 1–9. 10.3389/fnana.2015.00035 25873865 PMC4379933

[B55] Kuwabara N. DiCaprio R. A. Zook J. M. (1991). Afferents to the medial nucleus of the trapezoid body and their collateral projections. J. Comp. Neurol. 314, 684–706. 10.1002/cne.903140405 1816271

[B56] Lauer A. M. Dent M. L. Sun W. Xu-Friedman M. A. (2019). Effects of non-traumatic noise and conductive hearing loss on auditory system function. Neuroscience 407, 182–191. 10.1016/j.neuroscience.2019.01.020 30685543 PMC6513692

[B57] Lenn N. J. Reese T. S. (1966). The fine structure of nerve endings in the nucleus of the trapezoid body and the ventral cochlear nucleus. Am. J. Anat. 118, 375–389. 10.1002/aja.1001180205 5917192

[B58] Li H. S. Hultcrantz M. Borg E. (1993). Influence of age on noise-induced permanent threshold shifts in CBA/Ca and C57BL/6J mice. Audiology 32, 195–204. 10.3109/00206099309072935 8489480

[B59] Livingston G. Huntley J. Sommerlad A. Ames D. Ballard C. Banerjee S.. (2020). Dementia prevention, intervention and care: 2020 report of the Lancet Commission. Lancet 396, 413–446. 10.1016/S0140-6736(20)30367-6 32738937 PMC7392084

[B60] Loughrey D. G. Kelly M. E. Kelley G. A. Brennan S. Lawlor B. A. (2018). Association of age-related hearing loss with cognitive function, cognitive impairment and dementia a systematic review and meta-analysis. JAMA Otolaryngol. Head Neck Surg. 144, 115–126. 10.1001/jamaoto.2017.2513 29222544 PMC5824986

[B61] Masterton B. Jane J. A. Diamond I. T. (1967). Role of brainstem auditory structures in sound localization. I. Trapezoid body, superior olive and lateral lemniscus. J. Neurophysiol. 30, 341–359. 10.1152/jn.1967.30.2.341 4167210

[B62] McCullagh E. A. Salcedo E. Huntsman M. M. Klug A. (2017). Tonotopic alterations in inhibitory input to the medial nucleus of the trapezoid body in a mouse model of Fragile X syndrome. J. Comp. Neurol. 525, 3543–3562. 10.1002/cne.24290 28744893 PMC5615817

[B63] Mikaelian D. O. (1979). Development and degeneration of hearing in the C57/b16 mouse: relation of electrophysiologic responses from the round window and cochlear nucleus to cochlear anatomy and behavioral responses. Laryngoscope 89, 1–15. 10.1288/00005537-197901000-00001 423642

[B64] Mikaelian D. O. Warfield D. Norris O. (1974). Genetic progressive hearing loss in the c57/m6 mouse: relation of behaviorial responses to cochlear anatomy. Acta Otolaryngol. 77, 327–334. 10.3109/00016487409124632 4835632

[B65] Milinkeviciute G. Chokr S. M. Cramer K. S. (2021). Auditory brainstem deficits from early treatment with a csf1r inhibitor largely recover with microglial repopulation. eNeuro 8:ENEURO.0318-20.2021. 10.1523/ENEURO.0318-20.2021 33558268 PMC8009669

[B66] Milinkeviciute G. Henningfield C. M. Muniak M. A. Chokr S. M. Green K. N. Cramer K. S.. (2019). Microglia regulate pruning of specialized synapses in the auditory brainstem. Front. Neural Circuits 13, 1–19. 10.3389/fncir.2019.00055 31555101 PMC6722190

[B67] Moreno-García A. Kun A. Calero O. Medina M. Calero M. (2018). An overview of the role of lipofuscin in age-related neurodegeneration. Front. Neurosci. 12, 1–13. 10.3389/fnins.2018.00464 30026686 PMC6041410

[B68] Morgan J. L. Lichtman J. W. (2020). An individual interneuron participates in many kinds of inhibition and innervates much of the mouse visual thalamus. Neuron 106, e2468–e2481. 10.1016/j.neuron.2020.02.001 32142646 PMC7295017

[B69] Motta A. Berning M. Boergens K. M. Staffler B. Beining M. Loomba S.. (2019). Dense connectomic reconstruction in layer 4 of the somatosensory cortex. Science 366:eaay3134. 10.1126/science.aay3134 31649140

[B70] Nakajima Y. (1971). Fine structure of the medial nucleus of the trapezoid body of the bat with special reference to two types of synaptic endings. J. Cell Biol. 50, 121–134. 10.1083/jcb.50.1.121 5563443 PMC2108425

[B71] Parthasarathy A. Kujawa S. G. (2018). Synaptopathy in the aging cochlea: Characterizing early-neural deficits in auditory temporal envelope processing. J. Neurosci. 38, 7108–7119. 10.1523/JNEUROSCI.3240-17.2018 29976623 PMC6596096

[B72] Preibisch S. Saalfeld S. Tomancak P. (2009). Globally optimal stitching of tiled 3D microscopic image acquisitions. Bioinformatics 25, 1463–1465. 10.1093/bioinformatics/btp184 19346324 PMC2682522

[B73] Rodríguez-Contreras A. van Hoeve J. S. S. Habets R. L. P. Locher H. Borst J. G. G. (2008). Dynamic development of the calyx of Held synapse. Proc. Natl. Acad. Sci. U S A 105, 5603–5608. 10.1073/pnas.0801395105 18375766 PMC2291093

[B74] Rowland K. C. Irby N. K. Spirou G. A. (2000). Specialized synapse-associated structures within the calyx of Held. J. Neurosci. 20, 9135–9144. 10.1523/JNEUROSCI.20-24-09135.2000 11124991 PMC6773032

[B75] Rutherford M. A. von Gersdorff H. Goutman J. D. (2021). Encoding sound in the cochlea: from receptor potential to afferent discharge. J. Physiol. 599, 2527–2557. 10.1113/JP279189 33644871 PMC8127127

[B76] Sätzler K. Söhl L. F. Bollmann J. H. Borst J. G. G. Frotscher M. Sakmann B.. (2002). Three-dimensional reconstruction of a calyx of Held and its postsynaptic principal neuron in the medial nucleus of the trapezoid body. J. Neurosci. 22, 10567–10579. 10.1523/JNEUROSCI.22-24-10567.2002 12486149 PMC6758464

[B77] Schmidt H. Gour A. Straehle J. Boergens K. M. Brecht M. Helmstaedter M. (2017). Axonal synapse sorting in medial entorhinal cortex. Nature 549, 469–475. 10.1038/nature24005 28959971

[B78] Schneggenburger R. Forsythe I. D. (2006). The calyx of held. Cell Tissue Res. 326, 311–337. 10.1007/s00441-006-0272-7 16896951

[B79] Sergeyenko Y. Lall K. Liberman M. C. Kujawa S. G. (2013). Age-related cochlear synaptopathy: an early-onset contributor to auditory functional decline. J. Neurosci. 33, 13686–13694. 10.1523/JNEUROSCI.1783-13.2013 23966690 PMC3755715

[B80] Sierksma M. C. Slotman J. A. Houtsmuller A. B. Borst J. G. G. (2020). Structure-function relation of the developing calyx of Held synapse *in vivo*. J. Physiol. 598, 4603–4619. 10.1113/JP279976 33439501 PMC7689866

[B81] Sinclair J. L. Fischl M. J. Alexandrova O. Heβ M. Grothe B. Leibold C.. (2017). Sound-evoked activity influences myelination of brainstem axons in the trapezoid body. J. Neurosci. 37, 8239–8255. 10.1523/JNEUROSCI.3728-16.2017 28760859 PMC5566870

[B82] Singh M. Miura P. Renden R. (2018). Age-related defects in short-term plasticity are reversed by acetyl-L-carnitine at the mouse calyx of Held. Neurobiol. Aging 67, 108–119. 10.1016/j.neurobiolaging.2018.03.015 29656010 PMC5955853

[B83] Sommer I. Lingenhöhl K. Friauf E. (1993). Principal cells of the rat medial nucleus of the trapezoid body: an intracellular *in vivo* study of their physiology and morphology. Exp. Brain Res. 95, 223–239. 10.1007/BF00229781 8224048

[B84] Sonntag M. Englitz B. Kopp-Scheinpflug C. Rübsamen R. (2009). Early postnatal development of spontaneous and acoustically evoked discharge activity of principal cells of the medial nucleus of the trapezoid body: an *in vivo* study in mice. J. Neurosci. 29, 9510–9520. 10.1523/JNEUROSCI.1377-09.2009 19641114 PMC6666529

[B85] Spongr V. P. Flood D. G. Frisina R. D. Salvi R. J. (1997). Quantitative measures of hair cell loss in CBA and C57BL/6 mice throughout their life spans. J. Acoust. Soc. Am. 101, 3546–3553. 10.1121/1.418315 9193043

[B86] Svara F. N. Kornfeld J. Denk W. Bollmann J. H. (2018). Volume EM reconstruction of spinal cord reveals wiring specificity in speed-related motor circuits. Cell Rep. 23, 2942–2954. 10.1016/j.celrep.2018.05.023 29874581

[B87] Taberner A. M. Liberman M. C. (2005). Response properties of single auditory nerve fibers in the mouse. J. Neurophysiol. 93, 557–569. 10.1152/jn.00574.2004 15456804

[B88] Taschenberger H. Leão R. M. Rowland K. C. Spirou G. A. von Gersdorff H. (2002). Optimizing synaptic architecture and efficiency for high-frequency transmission. Neuron 36, 1127–1143. 10.1016/s0896-6273(02)01137-6 12495627

[B89] Terman A. Brunk U. T. (1998). Lipofuscin: mechanisms of formation and increase with age. APMIS 106, 265–276. 10.1111/j.1699-0463.1998.tb01346.x 9531959

[B90] Thomas C. I. Keine C. Okayama S. Satterfield R. Musgrove M. Guerrero-Given D.. (2019). Presynaptic mitochondria volume and abundance increase during development of a high-fidelity synapse. J. Neurosci. 39, 7994–8012. 10.1523/JNEUROSCI.0363-19.2019 31455662 PMC6786813

[B91] Vicencio-Jimenez S. Weinberg M. M. Bucci-Mansilla G. Lauer A. M. (2021). Olivocochlear Changes associated with aging predominantly affect the medial olivocochlear system. Front. Neurosci. 15, 1–16. 10.3389/fnins.2021.704805 34539335 PMC8446540

[B92] Wang H. Li B. Lu Y. Han K. Sheng H. Zhou J.. (2021). Real-time threshold determination of auditory brainstem responses by cross-correlation analysis. iScience 24:103285. 10.1016/j.isci.2021.103285 34765914 PMC8571499

[B93] Wang M. Zhang C. Lin S. Wang Y. Seicol B. J. Ariss R. W.. (2021). Biased auditory nerve central synaptopathy is associated with age-related hearing loss. J. Physiol. 599, 1833–1854. 10.1113/JP281014 33450070 PMC8197675

[B94] Willott J. F. (1986). Effects of aging, hearing loss and anatomical location on thresholds of inferior colliculus neurons in C57BL/6 and CBA mice. J. Neurophysiol. 56, 391–408. 10.1152/jn.1986.56.2.391 3760927

[B95] Wimmer V. C. Horstmann H. Groh A. Kuner T. (2006). Donut-like topology of synaptic vesicles with a central cluster of mitochondria wrapped into membrane protrusions: a novel structure-function module of the adult calyx of held. J. Neurosci. 26, 109–116. 10.1523/JNEUROSCI.3268-05.2006 16399677 PMC6674328

[B96] Xiao L. Michalski N. Kronander E. Gjoni E. Genoud C. Knott G.. (2013). BMP signaling specifies the development of a large and fast CNS synapse. Nat. Neurosci. 16, 856–864. 10.1038/nn.3414 23708139

[B97] Xiong Y. Z. Addleman D. A. Nguyen N. A. Nelson P. B. Legge G. E. (2022). Visual and auditory spatial localization in younger and older adults. Front. Aging Neurosci. 14, 1–12. 10.3389/fnagi.2022.838194 35493928 PMC9043801

[B98] Yu X. Wang Y. (2022). Tonotopic differentiation of presynaptic neurotransmitter-releasing machinery in the auditory brainstem during the prehearing period and its selective deficits in Fmr1 knockout mice. J. Comp. Neurol. 530, 1–22. 10.1002/cne.25406 36067267 PMC9588645

[B99] Zhang C. Beebe N. L. Schofield B. R. Pecka M. Burger R. M. (2021). Endogenous cholinergic signaling modulates sound-evoked responses of the medial nucleus of the trapezoid body. J. Neurosci. 41, 674–688. 10.1523/JNEUROSCI.1633-20.2020 33268542 PMC7842756

[B100] Zhang Y. Lin G. Wang Y. Xue N. Lin X. Du T.. (2022). Prestin derived OHC surface area reduction underlies age-related rescaling of frequency place coding. Hear. Res. 423:108406. 10.1016/j.heares.2021.108406 34933788

